# Prevalence and Diversity of *Bartonella* Species in Rodents from Georgia (Caucasus)

**DOI:** 10.4269/ajtmh.16-0041

**Published:** 2016-08-03

**Authors:** Lile Malania, Ying Bai, Lynn M. Osikowicz, Nikoloz Tsertsvadze, Guram Katsitadze, Paata Imnadze, Michael Kosoy

**Affiliations:** ^1^ General Bacteriology Laboratory, National Center for Disease Control and Public Health, Tbilisi, Republic of Georgia; ^2^ Bacterial Diseases Branch, Division of Vector-Borne Disease, Centers for Disease Control and Prevention, Fort Collins, Colorado

## Abstract

*Bartonella* infections are widespread and highly prevalent in rodents. Several rodent-associated *Bartonella* species have been related to human diseases. Recently, *Bartonella* species was reported as the etiology of a human case in the country of Georgia (Caucasus). However, information on *Bartonella* in rodents in Georgia is absent. Rodent hearts were collected from Georgia to investigate the presence and diversity of *Bartonella* species. *Bartonella* bacteria were cultured from 37.2% (16/43) of rodents examined, while *Bartonella* DNA was detected in 41.2% (28/68) of rodents by polymerase chain reaction targeting citrate synthase (*gltA*) gene. Sequences of *gltA* showed that rodents in this region harbored multiple *Bartonella* strains, including *Bartonella elizabethae*, *Bartonella tribocorum*, *Bartonella grahamii*, and an unknown genogroup. The first three *Bartonella* species, known to be rat-associated and human cases linked, were commonly observed in wood mice (*Apodemus* [*Sylvaemus*] *uralensis*) (5/8 positive with *B. elizabethae* and *B. tribocorum*) and social voles (*Microtus socialis*) (4/6 positive with *B. grahamii* and *B. elizabethae*) in this study. The frequent distribution of these *Bartonella* species suggests that they may contribute to unidentified clinical infections. The unknown genogroup was observed in 24 *Bartonella* isolates and/or DNA extracts from heart tissues, all of which were obtained from *Libyan jirds* (*Meriones libycus*). Further characterization of the bacterial cultures based on sequence analysis of four additional genes (*ftsZ*, *nuoG*, *rpoB*, and *ssrA*) supported that the jird-associated *Bartonella* strains comprise a distinct monophyletic clade. The impact of this bacterium on wildlife and human health needs to be determined.

## Introduction

Bacteria of the genus *Bartonella* are small, fastidious, and slow-growing Gram-negative aerobic rods. They parasitize erythrocytes and endothelial cells of a wide range of mammals^1^ and are usually host specific at different taxonomical levels.^2^–^5^ Currently, there are around 30 recognized species and/or subspecies within the genus that have been described from different vertebrate reservoirs. A number of *Bartonella* species have been associated with emerging diseases, involving a broad spectrum of clinical syndromes from self-limited cat-scratch disease (CSD) to potentially fatal diseases, such as endocarditis.^6^–^13^

*Bartonella* infections have been extensively studied in rodents of numerous species from various regions across the world, which demonstrated the very widespread and high prevalence of *Bartonella* species.^3^,^4^,^14^–^24^ Many rodent-associated *Bartonella* species have been described and at least five of them—*Bartonella elizabethae*, *Bartonella tribocorum*, *Bartonella grahamii*, *Bartonella vinsonii* subsp. *arupensis*, and *Bartonella washoensis* have been implicated as causative agents of human infections.^7^,^10^–^12^ *Bartonella* species usually are host specific. For example, *B. elizabethae* and *B. tribocorum* are specific to *Rattus* rats,^3^,^14^,^19^ whereas *B. washoensis* is specific to squirrels of family Sciuridae.^12^ These findings indicated the potential role of rodents as reservoirs for *Bartonella* species that could be pathogenic to humans. The close relation between rodents and humans throughout the world makes the study of rodent-borne *Bartonella* essential to determine the extent to which rodents may serve as sources of human infections. Although *Bartonella* infections are widely distributed and highly prevalent in rodents of different geographic regions, these bacteria have yet to be identified in rodents from Caucasus, including the country of Georgia. Diagnostics of infections in humans caused by *Bartonella* species are still limited in this country. However, a recent investigation of a human case with lymphadenopathy manifested as typical CSD in the country of Georgia has indicated that the agent was a *Bartonella* strain.^25^ The strain was closely related to both *B. tribocorum* and *B. elizabethae* rather than *Bartonella henselae*, the common agent of CSD, and the strain was identical to those frequently found in commensal rats in Israel.^23^ Such finding has indicated the need to investigate the diversity of *Bartonella* species in rodents in this region, considering a possibility that these species likely contribute to undiagnosed clinical human cases. The objectives of this study were to evaluate the presence of *Bartonella* species in rodents from Georgia and to investigate the genetic diversity of recovered *Bartonella* strains.

## Materials and Methods

### Study sites and rodent hearts collection.

Rodents were trapped within two field sites in May 2010. The first site is located in Dedoplistskaro municipality of Kakheti, the eastern region of Georgia bordering with Azerbaijan; the second site is located in Gardabani municipality of Kvemo Karli, the southeastern region of Georgia bordering with Azerbaijan and Armenia. Captured animals were identified morphologically to species, weighed, sexed, and dissected to collect hearts under sterile conditions. Of 60 rodents collected in Dedoplistskaro, 54 were identified as *Libyan jirds* (*Meriones libycus*) and six as social voles (*Microtus socialis*). Only eight rodents were sampled in Gardabani and all were identified as wood mice (*Apodemus* [*Sylvaemus*] *uralensis*). The collected rodent hearts were kept at −20°C until sent to U.S. CDC's laboratory in Fort Collins, CO, for testing.

### *Bartonella* culturing.

Rodent hearts (∼50 mg) were homogenized in 200 μL brain heart infusion broth supplemented with amphotericin B (final concentration 25 μg/mL) using Bullet Blender^®^ Gold homogenizer (Next Advance Inc., New York, NY) following heart protocol provided by the manufacturer. The homogenates (100 μL) were then plated on heart infusion agar supplemented with 10% rabbit blood and incubated in an aerobic condition with 5% CO_2_ at 35°C for up to 4 weeks. Plates were monitored for bacterial growth twice a week after initial plating. Bacterial colonies were presumptively identified as probable *Bartonella* species based on their morphological characteristics. A single colony from each plate was picked and streaked onto secondary agar plates for another 4–5 days of incubation under the same condition. If colonies on the same plate looked different, a single colony for each appearance is subcultured separately. Pure cultures were harvested in 10% glycerol.

### Polymerase chain reaction verification of *Bartonella* cultures by *gltA*.

*Bartonella* cultures were confirmed by polymerase chain reaction (PCR) amplifying a 378-bp fragment in the citrate synthase (*gltA*) gene using *Bartonella*-specific primers BhCS781.p and BhCS1137.n.^26^ Crude DNA extracts were obtained by heating a heavy suspension of the cultures at 95°C for 10 minutes followed by centrifugation of 30 seconds at 1,000 rpm. Supernatant was removed to a new tube and used as the template for down streaming analysis. Each PCR was conducted in a C1000 Touch Thermal Cycler (Bio-Rad, Hercules, CA) using the following program parameters: initial denaturing at 95°C for 3 minutes; 35 cycles of 95°C for 30 seconds, 51°C for 30 seconds, and 72°C for 30 seconds; and ending with 72°C for 7 minutes. PCR products were analyzed for the presence of amplicons of the appropriate size by electrophoresis in 1.5% agar gels containing ethidium bromide. Positive and negative controls were included in each PCR assay to evaluate the presence of appropriately sized amplicons and contamination, respectively.

### Molecular detection of *Bartonella* DNA in heart tissues.

Direct PCR was performed on DNA extracted from the remaining heart homogenates using QIAamp DNA Mini Kit (QIAGEN, Valencia, CA) following the tissue protocol provided by the manufacturer. Primers CS443F^16^ and CS1210R^22^ were used to generate a 747-bp fragment in *gltA* that incorporates the region amplified by primers BhCS781.p and BhCS1137.n used for *Bartonella* cultures. All conditions were the same as the first PCR assay but using the following parameters: initial denaturing at 94°C for 2 minutes; 38 cycles of 94°C for 30 seconds, 48°C for 30 seconds, and 72°C for 2 minutes; and ending with 72°C for 7 minutes.

### Sequencing.

*Bartonella gltA*–positive PCR products from both PCR assays were purified with the QIAquick PCR Purification Kit (QIAGEN, Germantown, MD) according to the manufacturer's instructions and sequenced in both directions using an Applied Biosystems Model 3130 Genetic Analyzer (Applied Biosystems, Foster City, CA). Sequencing reactions were carried out in a C1000 Touch Thermal Cycler using the same primers as the initial PCR assays at a concentration of 1.6 μM. Cycle parameters for the sequencing reactions were 96°C for 1 minute, followed by 24 cycles of 96°C for 10 seconds, 50°C for 5 seconds, and 60°C for 4 minutes.

### Phylogenetic analysis.

The resulting *gltA* sequences were analyzed using Lasergene sequence analysis software Version 12 (DNASTAR, Madison, WI) to determine consensus of sequences for the amplified region of the *gltA*. The Clustal W program within MegAlign of Lasergene was used to align and compare homology of *Bartonella gltA* sequences obtained from all samples in this study among themselves and with other known *Bartonella* species or genotypes available in the public domain for an initial *Bartonella* species identification. The neighbor-joining method by Kimura's two-parameter distance method and bootstrap calculation was carried out with 1,000 replicates. Newly identified variants from this study were submitted to GenBank and assigned with unique accession numbers (KT327027–KT327033).

### Characterization of the *L. jird*-originated *Bartonella* strains.

Comparing multiple genes can greatly increase a discriminatory power in genetic analysis of bacterial population.^27^ Based on *gltA* sequences identification, the novel *Bartonella* strains isolated from *L. jird* (*n* = 12) were further analyzed using four additional genes, including cell division (*ftsZ*), nicotinamide adenine dinucleotide dehydrogenase gamma-subunit (*nuoG*), RNA polymerase beta subunit (*rpoB*), and transfer-messenger RNA (*ssrA*), following the protocols published elsewhere.^5^ Phylogenetic analyses were performed for each gene as done for *gltA* to compare the homology between the isolates and described *Bartonella* species and genotypes. Newly identified variants for each gene were deposited in GenBank with accession numbers KT327034–KT327035 (*ftsZ*), KT327036–KT327037 (*nuoG*), KT327038–KT327039 (*ssrA*), and KT327040–KT327041 (*rpoB*).

### Ethics statement.

This research was conducted in compliance with the Animal Welfare Act and other Federal statutes and regulations related to animals and experiments involving animals and adheres to principles stated in the Guide for the Care and Use of Laboratory Animals, National Research Council Publication (1996 edition). All procedures involving animals were conducted under animal use protocols approved by the Institutional Animal Care and Use Committees of the National Center for Disease Control and Public Health, Tbilisi, Georgia.

## Results

### *Bartonella* culture.

Among the samples taken from 68 rodent hearts and cultured individually on agar plates, 25 (19 *L. jirds* and 6 social voles) were overgrown by fast growing non-*Bartonella* microorganisms which meant no conclusion could be made whether these organs were infected. From the other 43 remaining heart samples, which consisted of 8 wood mice and 35 *L. jirds*, *Bartonella* cultures were obtained from 16 individuals, including 4 wood mice and 12 jirds (Table 1). In total, 17 isolates were obtained from these 16 rodents as a result of 2 isolates were cultured from 1 wood mouse from which *Bartonella*-like colonies with differing morphologies were observed during the primary plating. All isolates were confirmed as *Bartonella* species by PCR with *gltA*. Therefore, the overall prevalence of *Bartonella* infections by culturing was 37.2% (16/43), with 50% (4/8) in wood mice and 34.3% (12/35) in *L. jirds*. All of the obtained isolates were further analyzed by sequencing.

### *Bartonella* prevalence estimated by PCR in heart tissues.

Molecular detection by PCR for all 68 rodents identified that 28 rodent hearts were positive for *Bartonella* using *gltA* primers. This included 2 wood mice, 22 *L. jirds*, and 4 social voles (Table 1). Overall prevalence by molecular detection was 41.2% (28/68), showing no significant difference from the prevalence estimated by culturing (χ^2^ = 0.105, *P* > 0.05).

### Genetic identification of *Bartonella* species based on the *gltA* analysis.

**Figure 1.**
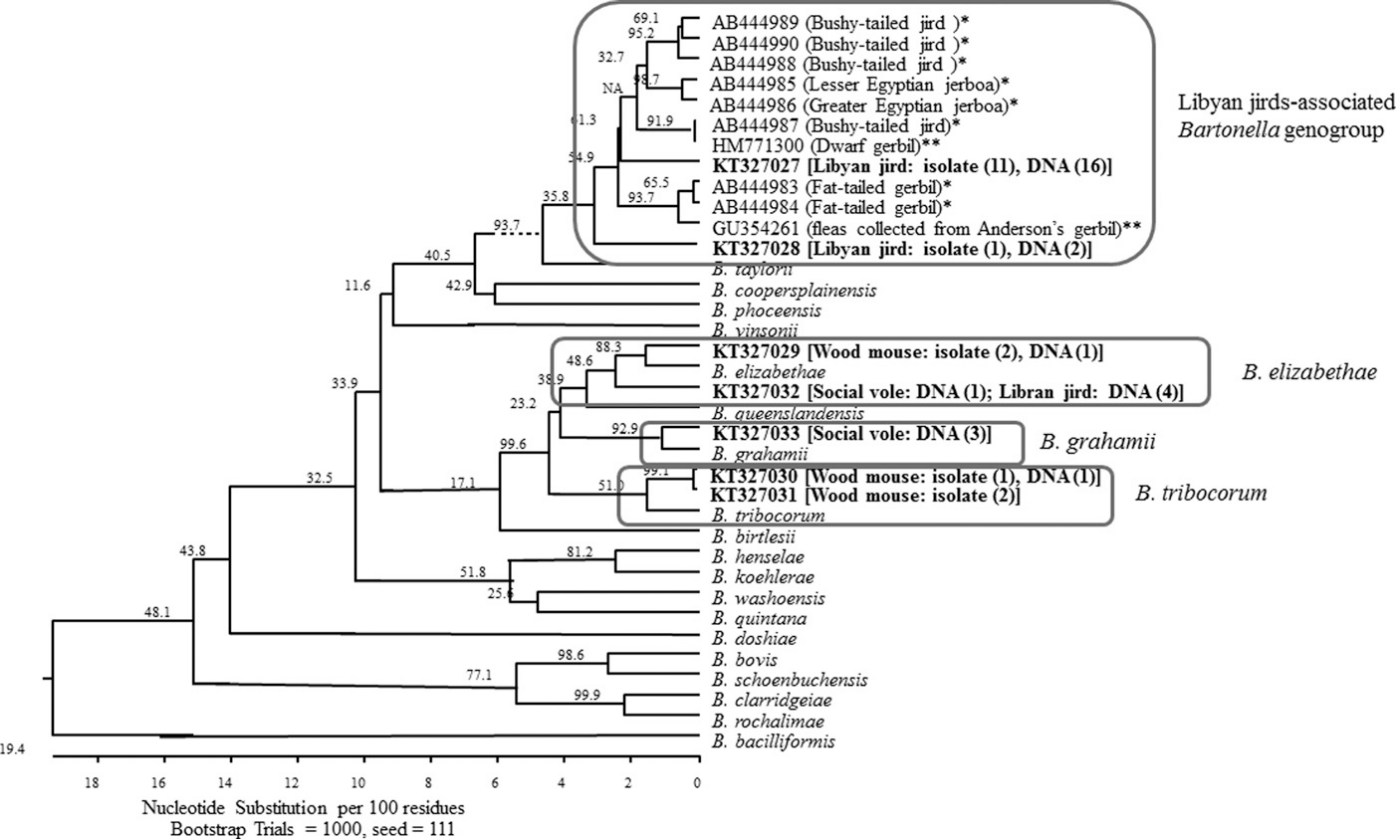
Phylogenetic relationships between the *Bartonella* genotypes detected in *Libyan jirds* (*Meriones libycus*) and other rodents from Georgia and some reference *Bartonella* species and related *Bartonella* variants based on partial sequences of *gltA*. Seven *Bartonella* genotypes were identified in the Georgian rodents. Each genotype is indicated by its GenBank accession number in bold and is followed by rodent species and number of identical isolates and DNA obtained from the host species in brackets and parenthesis. The genotypes belonged to *Bartonella elizabethae*, *Bartonella tribocorum*, *Bartonella grahamii*, and an unknown genogroup (square circled clades). The unknown genogroup consisted of two genotypes identified in the *L. jird* in the study and several genotypes previously described in different species of rodents and fleas by Inoue (*) and Morick (**). The phylogenetic tree was constructed by the neighbor-joining method, and bootstrap values were calculated with 1,000 replicates.

Sequencing analysis of *gltA* amplicons for all 17 isolates showed that the *Bartonella* strains circulating among the investigated rodents belonged to multiple groups, including *B. elizabethae*, *B. tribocorum*, and an unknown genogroup (Figure 1

). The five isolates cultured from wood mice belonged to either *B. elizabethae* (two isolates) or *B. tribocorum* (three isolates). The two isolates obtained from the same wood mouse each belonged to *B. elizabethae* and *B. tribocorum*. The strain of *B. tribocorum* was close to the variant FJ577651 previously reported in Israel rats^23^ or the human strain described in the Georgian case^25^ with similarity of 96.3%. The 12 isolates obtained from *L. jirds* were identical or close to each other and belong to 2 closely related variants (95.6% similarity). One variant (GenBank accession number KT327027) was the dominant and was identified in 11 of the 12 isolates, whereas another variant (GenBank accession number KT327028) was found only in one isolate. Both of the two jird-associated variants were similar to several variants reported from Japan^28^ and Israel.^29^,^30^ These variants were identified in different species of rodents, including dwarf gerbil (*Gerbillus nanus*), Lesser Egyptian jerboa (*Jaculus jaculus*), Greater Egyptian jerboa (*Jaculus orientalis*), fat-tailed gerbil (*Pachyuromys duprasi*), and bushy-tailed jird (*Sekeetamys calurus*), and fleas (*Synosterrnus leopatrae*) collected from Anderson's gerbil (*Gerbillus andersoni allenbyi*), with similarities ranging from 94.6% to 97.6%.^28^–^30^ Together, these variants formed a separate genogroup that is distant (> 7.6% distance) from all previously described *Bartonella* species or genotypes (Figure 1).

Sequences from all of the 28 *gltA*-positive DNA samples from rodent hearts were obtained. In addition to *B. elizabethae*, *B. tribocorum*, and the unknown genogroup as identified in the cultures, *B. grahamii* was identified in the heart tissues. Two sequences obtained from wood mice were close to either *B. elizabethae* (*N* = 1) or *B. tribocorum* (*N* = 1); the sequences obtained from four social voles belonged to *B. grahamii* with one exception for which the sequence belonged to *B. elizabethae*. Of the 22 *Bartonella*-positive *L. jirds*, 18 sequences belonged to the same novel genogroup identified in the cultures obtained from the same rodent species in this study, with the majority (16/18) being identical to the variant KT327027 (the dominant one), and the other 2 being identical to the variant KT327028. The last four *Bartonella*-positive *L. jirds* belonged to *B. elizabethae* (Figure 1).

### Characterization of the novel strain from the *L. jird*.

Analyses based on *gltA* sequences for both bacterial cultures and direct molecular detection in tissues have suggested that most *Bartonella* isolates/DNA detected in *L. jird* belong to a unique genogroup that is genetically distant from other known *Bartonella* species and/or genotypes. Further sequencing analysis of *ftsZ*, *nuoG*, *rpoB*, and *ssrA* amplicons for the 12 isolates cultured from *L. jird* demonstrated that 11 of the 12 isolates were identical between themselves for each of the marker genes used. The remaining isolate was close, but different from other cultures, with a divergence of 2.4%, 3.7%, 3.7%, and 1.7% for the *ftsZ*, *nuoG*, *rpoB*, and *ssrA* amplicon sequences, respectively. Phylogenetic analysis based on each of the targets demonstrated that the two variants were grouped together and comprised a distinct monophyletic clade with divergences of at least 7%, 7%, 5%, and 4% from all described *Bartonella* species or genotypes by *ftsZ*, *nuoG*, *rpoB*, and *ssrA*, respectively. These results suggested that the strains isolated from *L. jirds* are unique and likely represent a novel *Bartonella* species according to the definition of bacterial species.^31^

## Discussion

We herein report identification of *Bartonella* infections in rodents from the country of Georgia. Prevalence of *Bartonella* species in local rodents was found to be comparable to some previously investigated regions.^3^,^14^,^17^,^19^ Comparative analyses of partial sequences of *gltA* gene that has been shown to be reliable for distinguishing between closely related *Bartonella* genotypes^26^,^32^ showed that rodents in Georgia harbor multiple *Bartonella* species, including *B. elizabethae*, *B. tribocorum*, *B. grahamii*, and a presumably novel species that is associated with *L. jirds*.

*Bartonella elizabethae* and *B. tribocorum* are known human pathogens.^7^,^33^,^34^ Previous studies strongly supported that both *B. elizabethae* and *B. tribocorum* are associated with rats of the genus *Rattus*.^3^,^14^,^19^,^35^,^36^ Unexpectedly, in this study, both *B. elizabethae* and *B. tribocorum* were identified with obtaining cultures from wood mice and detecting specific DNA in tissues of the sylvatic rodents. Moreover, both species were observed in one wood mouse, suggesting the existence of coinfection. Our findings are very important in providing evidence of wide distribution of *B. elizabethae* and *B. tribocorum* in Georgian rodents. The identification of the Georgian human case with lymphadenopathy as a *Bartonella* strain closely related to both *B. tribocorum* and *B. elizabethae* and identical to strains commonly found in commensal rats in Israel^23^,^25^ suggests that commensal rats are a likely source of this case. However, identification of related strain in the wild rodents in Georgia suggests that those also should be considered as potential sources of *Bartonella* infections in people having a contact with rodents. Investigation of the role of *B. elizabethae* and *B. tribocorum* as a source for cases of noncultivable bacterial endocarditis and febrile illnesses of unknown etiology in Georgia is required to estimate their epidemiological importance.

In contrast to this report, previous studies of *Bartonella* species in *Apodemus* mice conducted in China, Korea, Japan, Russia, and other regions have shown that these mice carry other *Bartonella* species rather than *B. elizabethae* or *B. tribocorum*.^3^,^24^,^37^ Rats of genus *Rattus* have not been examined in this study, but our observation may suggest that sylvatic rodent species in some natural habitats of Georgia can serve as additional reservoirs for these *Bartonella* species. This suggests the need for further investigations on distribution of *Bartonella* species in *Rattus* rats across Georgia. Multilocus characterization of the obtained strains is needed to understand relations between *Bartonella* species carried by commensal and sylvatic rodents in Caucasus.

The majority of the examined *L. jirds* were infected with similar or closely related *Bartonella* strains. These strains are clustered into a distinct phylogenetic lineage with variants previously identified in different species of gerbils and fleas collected from Anderson's gerbil.^28^–^30^ Although the range of similarities between the *gltA* fragments among these variants (94.6–97.6%) was at borderline proposed for definition of bacterial species,^31^ they likely belong to the same species complex according to recently proposed classification of *Bartonella* species.^38^ It is not surprising since *L. jirds*, dwarf gerbil, Lesser Egyptian jerboa, Greater Egyptian jerboa, fat-tailed gerbil, and bushy-tailed jird all belong to the gerbil subfamily. The results of multi-locus sequence typing analyses based on sequences of five genes (*ftsZ*, *gltA*, *nuoG*, *rpoB*, and *ssrA*) support the opinion that the strains associated with gerbils form a single unique genogroup. Although some bacteria belonging to this group were reported from Japan,^28^ those animals were not native there, but were imported to Japan from the Middle and Near East (Egypt and Pakistan), as these animals were selected from pet stores for the investigation. These observations suggested that this lineage is likely associated with gerbils that are distributed in the region of Mediterranean and around and presumably have a wide distribution among various related species of jirds, gerbils, and jerboas. The high percentage of *Bartonella*-positive individuals among *L. jirds* observed in Georgia suggested that *L. jirds* likely serve as the major host for this *Bartonella* lineage. More investigations are warranted to determine the impact of this bacterium on wildlife and humans.

## Figures and Tables

**Table 1:** *Bartonella* species in rodents from Georgia (Caucasus), May 2010

Species	Culture			Molecular detection (*gltA*)			*Bartonella* species* (*n*)
	No. tested	No. positive	Prevalence (%)	No. tested	No. positive	Prevalence (%)	
*Apodemus (Sylvaemus) uralensis* (the wood mouse)	8	4†	50	8	2	25	*B. e* (2), *B. t* (3)
*Meriones libycus* (the *Libyan jird*)	35‡	12	34.3	54	22	42.6	Jird-associated *Bartonella* sp. (22)
*Microtus socialis* (the social vole)	0‡	0	not applicable	6	4	83.3	*B. e* (1), *B. g* (3)
Total	43	16	37.2	68	28	41.2	

* *B. e* = *Bartonella elizabethae*; *B. t* = *Bartonella tribocorum*; *B. g* = *Bartonella grahamii.*

† There were four positive individuals but five isolates due to multiple isolates from one individual.

‡ Nineteen *L. jirds* and six social voles were contaminated, and so were excluded from culture.

## References

[1] Dehio C (2001). *Bartonella* interactions with endothelial cells and erythrocytes. Trends Microbiol.

[2] Birtles RJ, Harrison TG, Molyneux DH (1994). *Grahamella* in small woodland mammals in the U.K–isolation, prevalence and host-specificity. Ann Trop Med Parasitol.

[3] Ying B, Kosoy MY, Maupin GO, Tsuchiya KR, Gage KL (2002). Genetic and ecologic characteristics of *Bartonella* communities in rodents in southern China. Am J Trop Med Hyg.

[4] Bai Y, Calisher CH, Kosoy MY, Root JJ, Doty JB (2011). Persistent infection or successive reinfection of deer mice with *Bartonella vinsonii* subsp. *arupensis*. Appl Environ Microbiol.

[5] Bai Y, Malania L, Castillo DA, Moran D, Boonmar S, Chanlun A, Suksawat F, Maruyama S, Knobel D, Kosoy M (2013). Global distribution of *Bartonella* infections in domestic bovine and characterization of *Bartonella bovis* strains using multi-locus sequence typing. PLoS One.

[6] Welch DF, Pickett DA, Slater LN, Steigerwalt AG, Brenner DJ (1992). *Rochalimaea henselae* sp. nov., a cause of septicemia, bacillary angiomatosis, and parenchymal bacillary peliosis. J Clin Microbiol.

[7] Daly JS, Worthington MG, Brenner DJ, Moss CW, Hollis DG, Weyant RS, Steigerwalt AG, Weaver RE, Daneshvar MI, O'Connor SP (1993). *Rochalimaea elizabethae* sp. nov. isolated from a patient with endocarditis. J Clin Microbiol.

[8] Anderson BE, Neuman MA (1997). *Bartonella* spp. as emerging human pathogens. Clin Microbiol Rev.

[9] Kerkhoff FT, Bergmans AM, van Der Zee A, Rothova A (1999). Demonstration of *Bartonella grahamii* DNA in ocular fluids of a patient with neuroretinitis. J Clin Microbiol.

[10] Welch DF, Carroll KC, Hofmeister EK, Persing DH, Robison DA, Steigerwalt AG, Brenner DJ (1999). Isolation of a new subspecies, *Bartonella vinsonii* subsp. *arupensis*, from a cattle rancher: identity with isolates found in conjunction with *Borrelia burgdorferi* and *Babesia microti* among naturally infected mice. J Clin Microbiol.

[11] Kosoy M, Murray M, Gilmore RD, Bai Y, Gage KL (2003). *Bartonella* strains from ground squirrels are identical to *Bartonella washoensis* isolated from a human patient. J Clin Microbiol.

[12] Kosoy M, Bai Y, Sheff K, Morway C, Baggett H, Maloney SA, Boonmar S, Bhengsri S, Dowell SF, Sitdhirasdr A, Lerdthusnee K, Richardson J, Peruski LF (2010). Identification of *Bartonell*a infections in febrile human patients from Thailand and their potential animal reservoirs. Am J Trop Med Hyg.

[13] Chomel BB, Boulouis HJ, Breitschwerdt EB (2004). Cat scratch disease and other zoonotic *Bartonella* infections. J Am Vet Med Assoc.

[14] Castle K, Kosoy M, Lerdthusnee K, Phelan L, Bai Y, Gage KL, Leepitakrat W, Monkanna T, Khlaimanee N, Chandranoi K, Jones JW, Coleman RE (2004). Prevalence and diversity of *Bartonella* in rodents of northern Thailand: a comparison with *Bartonella* in rodents from southern China. Am J Trop Med Hyg.

[15] Pretorius AM, Beati L, Birtles RJ (2004). Diversity of *Bartonellae* associated with small mammals inhabiting free state province, South Africa. Int J Syst Evol Microbiol.

[16] Jardine C, Appleyard G, Kosoy MY, McColl D, Chirino-Trejo M, Wobeser G, Leighton FA (2005). Rodent-associated *Bartonella* in Saskatchewan, Canada. Vector Borne Zoonotic Dis.

[17] Bai Y, Montgomery SP, Sheff KW, Chowdhury MA, Breiman RF, Kabeya H, Kosoy MY (2007). *Bartonella* strains in small mammals from Dhaka, Bangladesh, related to *Bartonella* in America and Europe. Am J Trop Med Hyg.

[18] Inoue K, Maruyama S, Kabeya H, Yamada N, Ohashi N, Sato Y, Yukawa M, Masuzawa T, Kawamori F, Kadosaka T, Takada N, Fujita H, Kawabata H (2008). Prevalence and genetic diversity of *Bartonella* species isolated from wild rodents in Japan. Appl Environ Microbiol.

[19] Bai Y, Kosoy MY, Lerdthusnee K, Peruski LF, Richardson JH (2009). Prevalence and genetic heterogeneity of *Bartonella* strains cultured from rodents from 17 provinces in Thailand. Am J Trop Med Hyg.

[20] Gundi VA, Taylor C, Raoult D, La Scola B (2009). *Bartonella rattaustraliani* sp. nov., *Bartonella queenslandensis* sp. nov. and *Bartonella coopersplainsensis* sp. nov., identified in Australian rats. Int J Syst Evol Microbiol.

[21] Gundi VA, Billeter SA, Rood MP, Kosoy MY (2012). *Bartonella* spp. in rats and zoonoses, Los Angeles, California, USA. Emerg Infect Dis.

[22] Halliday JE, Knobel DL, Agwanda B, Bai Y, Breiman RF, Cleaveland S, Njenga MK, Kosoy M (2015). Prevalence and diversity of small mammal-associated *Bartonella* species in rural and urban Kenya. PLoS Negl Trop Dis.

[23] Harrus S, Bar-Gal GK, Golan A, Elazari-Volcani R, Kosoy MY, Morick D, Avidor B, Baneth G (2009). Isolation and genetic characterization of a *Bartonella* strain closely related to *Bartonella tribocorum* and *Bartonella elizabethae* in Israeli commensal rats. Am J Trop Med Hyg.

[24] Kim KS, Inoue K, Kabeya H, Sato S, Takada T, Pangjai D, Chiu SH, Fujita H, Kawabata H, Takada N, Kariwa H, Maruyama S (2016). Prevalence and diversity of *Bartonella* species in wild small mammals in Asia. J Wildl Dis.

[25] Kandelaki G, Malania L, Bai Y, Chakvetadze N, Katsitadze G, Imnadze P, Nelson C, Harrus S, Kosoy M (2016). Human lymphadenopathy caused by a ratborne *Bartonella* strain, Tbilisi, Country of Georgia. Emerg Infect Dis.

[26] Norman AF, Regnery R, Jameson P, Greene C, Krause DC (1995). Differentiation of *Bartonella*-like isolates at the species level by PCR-restriction fragment length polymorphism in the citrate synthase gene. J Clin Microbiol.

[27] Chaloner GL, Ventosilla P, Birtles RJ (2011). Multi-locus sequence analysis reveals profound genetic diversity among isolates of the human pathogen *Bartonella bacilliformis*. PLoS Negl Trop Dis.

[28] Inoue K, Maruyama S, Kabeya H, Hagiya K, Izumi Y, Une Y, Yoshikawa Y (2009). Exotic small mammals as potential reservoirs of zoonotic *Bartonella* spp. Emerg Infect Dis.

[29] Morick D, Krasnov BR, Khokhlova IS, Shenbrot GI, Kosoy MY, Harrus S (2010). *Bartonella* genotypes in fleas (insecta: siphonaptera) collected from rodents in the negev desert, Israel. Appl Environ Microbiol.

[30] Morick D, Krasnov BR, Khokhlova IS, Gottlieb Y, Harrus S (2011). Investigation of *Bartonella* acquisition and transmission in *Xenopsylla ramesis* fleas (Siphonaptera: Pulicidae). Mol Ecol.

[31] La Scola B, Liang Z, Zeaiter Z, Houpikian P, Grimont PA, Raoult D (2002). Genotypic characteristics of two serotypes of *Bartonella henselae*. J Clin Microbiol.

[32] Birtles RJ, Raoult D (1996). Comparison of partial citrate synthase gene *(gltA)* sequences for phylogenetic analysis of *Bartonella* species. Int J Syst Bacteriol.

[33] Comer JA, Flynn C, Regnery RL, Vlahov D, Childs JE (1996). Antibodies to *Bartonella* species in inner-city intravenous drug users in Baltimore, MD. Arch Intern Med.

[34] O'Halloran HS, Draud K, Minix M, Rivard AK, Pearson PA (1998). Leber's neuroretinitis in a patient with serologic evidence of *Bartonella elizabethae*. Retina.

[35] Heller R, Riegel P, Hansmann Y, Delacour G, Bermond D, Dehio C, Lamarque F, Monteil H, Chomel B, Piémont Y (1998). *Bartonella tribocorum* sp. nov., a new *Bartonella* species isolated from the blood of wild rats. Int J Syst Bacteriol.

[36] Ellis BA, Regnery RL, Beati L, Bacellar F, Rood M, Glass GG, Marston E, Ksiazek TG, Jones D, Childs JE (1999). Rats of the genus *Rattus* are reservoir hosts for pathogenic *Bartonella* species: an Old World origin for a New World disease?. J Infect Dis.

[37] Mediannikov O, Ivanov L, Zdanovskaya N, Vysochina N, Fournier PE, Tarasevich I, Raoult D (2005). Molecular screening of *Bartonella* species in rodents from the Russian far east. Ann N Y Acad Sci.

[38] Kosoy M, Hayman DT, Chan KS (2012). Bartonella bacteria in nature: where does population variability end and a species start?. Infect Genet Evol.

